# A neglected causative agent in diabetic foot infection: a retrospective evaluation of 13 patients with fungal etiology

**DOI:** 10.3906/sag-1809-74

**Published:** 2019-02-11

**Authors:** Anıl Murat ÖZTÜRK, Meltem Işıkgöz TAŞBAKAN, Dilek Yeşim METIN, Can YENER, Serhat UYSAL, Ilgın YILDIRIM ŞIMŞIR, İlgen ERTAM, Hüsnü PULLUKÇU, Bilgin ARDA, Sevki ÇETINKALP

**Affiliations:** 1 Department of Orthopedics and Traumatology, Faculty of Medicine, Ege University, İzmir Turkey; 2 Department of Clinical Microbiology and Infectious Diseases, Faculty of Medicine, Ege University, İzmir Turkey; 3 Department of Clinical Microbiology, Faculty of Medicine, Ege University, İzmir Turkey; 4 Department of Clinical Microbiology and Infectious Diseases, Kanuni Training and Research Hospital, Trabzon Turkey; 5 Division of Endocrinology, Department of Internal Diseases, Faculty of Medicine, Ege University, İzmir Turkey; 6 Department of Dermatology, Faculty of Medicine, Ege University, İzmir Turkey

**Keywords:** Invasive fungal infection, foot infection, amputation, diabetic foot ulcer

## Abstract

**Background/aim:**

Clinicians often neglect fungal infections and do not routinely investigate deep tissue from the wound for fungal culture and sensitivity due to insufficient information in the literature. In this study, we aimed to evaluate fungal etiology of invasive fungal diabetic foot which is rarely reported in the literature.

**Materials and methods:**

The patients who were unresponsive to antibiotic therapy and those with positive fungal in bone or deep tissue culture were enrolled in the study. Detailed hospital records were retrieved for demographics and clinical features.

**Results:**

A total of 13 patients who were diagnosed with invasive fungal diabetic foot (ten females, three males, mean age 59.8 ± 9 years) were included. All of the patients had type-2 diabetes mellitus. Eleven (84.6%) patients had mixed infection. The most common cause of fungal infections of diabetic foot ulcers was the *Candida* species. Ten (76.9%) patients underwent amputation, two (15.4%) patients refused amputation, and one patient died before surgery.

**Conclusion:**

Invasive fungal infections may also be a causative pathogen in deep tissue infections. Therefore, fungal pathogens should be considered in patients unresponsive to long-term antibiotic therapy. Early detection of fungal infections in high-risk individuals is critical for the prevention of severe consequences such as foot amputation.

## 1. Introduction

Diabetes mellitus (DM) is the most common endocrine metabolic disease, and its prevalence has been increasing steadily all over the world with recently diagnosed cases (1,2). The most significant and devastating complications of diabetes are foot ulcers and other foot problems causing major morbidity and mortality in patients with DM. Diabetes mellitus generally affects the lower extremities more than the upper extremities, leading to death in some cases. The treatment period of the ulcers lasts for a long time, and active ulcers can lead to limb amputation due to poor treatment (3–7). Diabetic foot complications are the most common causes of nontraumatic lower extremity amputations and are associated with a five-year mortality rate of 43% to 55%, which is even higher than some common types of cancer such as Hodgkin’s disease, breast and prostate cancers (8–10).

Several pathogenic abnormalities, such as intrinsic flaws in the blood supply, angiogenesis, and other extrinsic factors, such as infections and continued trauma, contribute to failure of recovery from diabetic foot ulcers (11,12). Organisms causing diabetic foot infection (DFI) are not often identified completely by clinical laboratories. Although recent articles state that DFIs are usually polymicrobial, few papers from the literature report a low incidence of fungal isolation from the diabetic foot wound. Despite current treatment modalities for nonhealing diabetic foot ulcers, following systemic antifungal therapy will lead to improvement (13–21).

In spite of the evidence for fungal infection in diabetic foot lesions, clinicians and surgeons treat diabetic foot wounds as if they could only be bacterial infections and treat them with antibacterial agents. They often neglect fungal infections and do not routinely investigate deep tissue from the wound bed for fungal culture and sensitivity due to insufficient information in the literature. In this study, we aimed to evaluate fungal etiology of invasive fungal DFI which is rarely reported in the literature.

## 2. Materials and methods

### 2.1. Subjects

We reviewed the medical records of patients diagnosed with DFI and admitted to the Diabetic Foot Care Center of Ege University, Faculty of Medicine between March 2013 and May 2018.

The patients who were unresponsive to antibiotic therapy and those with positive fungal in bone or deep tissue culture were enrolled in the study. Detailed hospital records were retrieved for age, sex, symptoms, duration of diabetes, presence of osteomyelitis, tissue culture, PEDIS infection classification scores, presence of significant vascular stenosis, microbiological data, HbA1c levels, cause of trauma, wound localization, antibiotic therapy, surgical treatment, total hospitalization time, and presence of comorbid diseases. 

### 2.2. Fungal culture

Bone or deep tissue samples were crushed into small pieces and cultured on a Sabouraud Dextrose Agar (SDA) medium and incubated at 26 °C and 35 °C for 10 days. The yeast colonies were identified by conventional methods (germ tube and their micromorphological appearance on corn meal agar), ID32C (bioMérieux-France) carbohydrate assimilation feature, and matrix-assisted laser desorption ionization time of flight mass spectrometry (MALDI TOF MS). Filamentous fungi were identified as genus based on their appearance on media and microscopy using lactophenol cotton blue.

Ethics committee approval was obtained from the Research Ethics Committee of Ege University, İzmir, Turkey (approval No: 13-1.1/8).

## 3. Results

Bone or deep tissue cultures were positive in 13 patients who were diagnosed with diabetic foot infection (ten females, three males, mean age 59.8 ± 9 years). Age, sex, diabetes type, HbA1c level, CRP, osteomyelitis, vasculopathy, neuropathy, response to antibacterial therapy, wound characteristics, treatment types and results, and bacteriology and mycology of the ulcers were summarized in Table. Six patients had a history of prior diabetic foot infection and decreased lower extremity arterial perfusion. 

One of the patients refused amputation; therefore, only antifungal therapy was given. Five of the patients received antifungal therapy in addition to surgical treatment. Since two patients had died of cerebrovascular pathology before the culture results were available, antifungal therapy could not be started. Despite amputation, five patients had fungal growth in the culture. In spite of fungal growth in the culture, antifungal therapy was not given to these five patients thanks to adequate and clean surgical wound area at amputation level. Mixed infections were observed in 11 of 13 patients and pure fungal infections in two. The most common cause of fungal infections of diabetic foot ulcers was the *Candida* species (Table). 

**Table 1:** Demographics and characteristics of the patients. Tx: transplantation, CRF: Chronic renal failure, HT: Hypertension, RA: Romatoid arthritis, CRP: C reactive protein.

	Sex/age	Type of DM,year of diagnosis	HbA1c(%-mmol/mol)	CRP	Osteomyelitis	Vasculopathy	Neuropathy	Response toantibacterial therapy	Surgery,type of surgery	Bacteriologyof ulcer	Mycologyof ulcer	Result of the treatment
1	F/79	Type 2,10	6.1–43	13.69	Yes	Yes	Yes	No	Yes, below-knee amputation	Hafnia alvei	Candida lipolytica	Despite fluconazole therapy, rapidly progressed to amputation
2	F/53	Type 2,11	13.4–123	23.97	Yes	No	Yes	Yes, partially	Yes,transmetatarsal amputation	Pseudomonas aeruginosa,	C. glabrata	Despite amphotericin Btherapy, rapidly progressed to amputation
3	M/63	Type 2,10	13.3–122	26.2	Yes	Yes	No	No	Yes, above-knee amputation	P. aeruginosa,Corynebacterium striatum	C. albicans	Amputation performedwithout further culture
4	M/49	Type 2,1	13.0–119	11.96	Yes	Yes	Yes	No	Yes,toe amputation	P. aeruginosa	C. albicans	Amputation performed without further culture
5	M/64	Type 2,16	No	9,36	Yes	Yes	Yes	Yes, partially	Yes,Ray’s amputation	-	C. albicans	Amputation performed without further culture
6	M/61	Type 2,12	No	21.43	Yes	Yes	No	No	Yes,transmetatarsal amputation	P. aeruginosa	Fusarium spp.	Amputation performed without further culture
7	M/68	Type 2,19	6.2–44	9.16	Yes	No	Yes	Yes, partially	Yes,transmetatarsal amputation	-	C. parapsilosis	Patient died before culture results
8	F/50	Type 2,8	12.0–108	19.23	Yes	Yes	No	No	No	Streptococcus agalactiae	C. glabrata	After amphotericin B therapyCaspofungin, Healed
9	M/63	Type 2,3	7.3–56	23.30	No	Yes	Yes	Yes, partially	Yes, Ray’s amputation	Klebsiella oxytoca, Enterobacter cloacae	C. krusei	Amputation performed without further culture
10	M/53	Type 2,23	No	32.86	yes	Yes	Yes	Yes, partially	No	Pseudomonas aeruginosa,Acinetobacter baumannii	C. glabrata	Patient died before culture results
11	M/54	Type 2,15	9.0–75	1.0	No	Yes	Yes	Yes, partially	Yes, only debridment	Staphylococcus aureus	C.albicans	After fluconazole therapy,Healed
12	M/68	Type 2,10	7.9–63	16.63	Yes	Yes	Yes	Yes, partially	Yes, toe amputation	Staphylococcus aureus, Providencia stuartii	Trichosporon asahii,Fusarium solani	After itraconazole therapy,Healed
13	M/56	Type 2,20	6.2–44	4.7	Yes	Yes	Yes	No	No	Alcaligenes faecalis, Proteus vulgaris	Fusarium spp.	After liposomal amphoterisin B, Healed

## 4. Discussion

Diabetes mellitus is the most common endocrine metabolic disease and its incidence is increasing rapidly. Foot ulcers and their complications are major causes of morbidity and mortality as are other complications in people with DM (12,22). Little is known about invasive fungal infections in the literature because there are only a few studies on osteomyelitis caused by fungal pathogens. The reasons are that clinicians do not routinely request fungal culture and sensitivity test for deep tissue from the wound bed due to lack of literature support and that fungi are neglected as causative agents after cultivating bacteria in the cultures. Patients with poorly-controlled diabetes are more prone to fungal infections (23). According to a brief overview of the literature, the rate of fungal isolation from diabetic foot ulcers varies from 5% to 27% (13–21,23). Chellan et al. (13) reported the incidence of fungal infections in patients with diabetic foot ulcer as 27.2%. The present study is retrospective and aims to reveal the fungal infections of diabetic foot ulcers analyzed from tissue biopsies in patients who did not recover despite proper antibacterial therapy. This is one of the large series that have been reported in the literature about invasive fungal infections as causative pathogens in diabetic foot infections.

Eckhard et al. (18) stated that fungal foot infection in patients with diabetes was correlated with age, sex, and duration of diabetes. Males were more frequently affected than females. Only HbA1c level was found to be significant in the incidence of fungal foot infection in the type-2 DM group. Although another study noted no difference in fungal foot infection rates among patients’ age, sex, depth of wound, duration of diabetes, it was mentioned that poor and inadequate glycemic control among diabetes patients is a risk factor for fungal infection (13). Raiesi et al. (23) compared skin and nail fungal infections with diabetic foot ulcers. Only the type of diabetes was significantly different in diabetic foot ulcers, and no significance was revealed in the average age of patients and the duration of diabetes. Skin and nail lesions were more common in type-2 DM patients whereas diabetic foot ulcers were more common in type-1 DM patients. Although the ratio of fungal infection was 19.1% in diabetic foot ulcers, it was 28% in patients with skin and nail lesions. In this study, all cases suffered from type-2 DM (duration of 1–23 years, mean duration: 12–15 years). Most of the patients were male, only three were female. The mean age was 60.1 (50–79).

Diabetic foot infections are multifactorial processes involving neuropathy, peripheral vascular disease, and susceptibility to infection, all contributing to tissue damage. Vasculopathy and neuropathy often lead to nonhealing ulcers coupled with recurrent soft-tissue infections and subsequent osteomyelitis of foot bones (24). In this study, 11 of the 13 patients had vasculopathy, 10 of the 13 patients had neuropathy, and eight patients had both vasculopathy and neuropathy. Osteomyelitis was present in all cases except for two. Although vasculopathy and neuropathy play a central role, osteomyelitis was not observed in those two patients who had vasculopathy and neuropathy. It is suggested that some factors disrupting skin integrity should also be included in the development of diabetic foot infections.

Diabetic foot infections are often polymicrobial (25). Acute infections are usually caused by gram-positive cocci (*Staphylococcus aureus*, B-hemolytic streptococci). However, gram-positive cocci and *Enterobacteriaceae*, diphtheroid species, *Pseudomonas aeruginosa*, other nonfermentative gram-negative bacilli and fungi are mostly observed in long-term nonhealing wounds, which are treated with long-term broad-spectrum antibiotic therapy. In the wounds, these are necrotic or ischemic and the most common causative agents are anaerobes (26–29). In line with the literature, concurrent bacterial infections were also detected in 11 of 13 patients with fungal diabetic foot infections. Seven patients responded to antibacterial therapy partially, whereas five patients did not benefit from it. In spite of the partial response to antibacterial therapy, six of them underwent surgical treatment. Despite the antifungal therapy, gram-negative bacteria accompanied fungi in the two patients who underwent amputation. Infection caused by the *Candida* species was observed only in two patients. Before the culture results were out, one of the patients had amputation and the other died. Four of six patients who had gram-negative bacterial infection also underwent amputation without culture results. Three of 13 patients, who were given antifungal therapy, recovered without surgery. Early initiation of therapy may reduce morbidity in cases when antibiotic treatment is not successful due to possible fungal infection.

In patients with diabetes, fungal foot infections like tinea pedis and onychomycosis are even more common inflammatory skin diseases compared to general population (23,26–31). The predisposing factors, which are high blood glucose, vasculopathy, neuropathy and various immunological disturbances, facilitate conditions for colonization of pathogen fungi, including *Candida* spp., *Dermatophytes*, *Malassezia*, *Mucorales*, *Aspergillus*, and *Fusarium *spp. (29). These fungi can also be caused by skin and soft tissue infections or onychomycosis in patients whose feet remain in moisture for a long time, or in cases of poor hygiene and trauma. Many studies show that fungal infections of the skin and nails including tinea infections are risk factors for cellulitis (31). The presence of superficial fungal foot infections could not be assessed and this was a limitation of our study. Many studies consistently identified fungal foot infection as a factor in the development of cellulitis, especially inter-digital rather than plantar and nail infections (31). Similar to our study, the most common pathogen in the literature is *Candida *spp. (13–21,23). *Candida* and other superficial fungal infections lead to a combination of higher humidity and thinner skin, which makes it more susceptible to infection (31). In DM, some pathogens other than bacteria like myiasis can cause delayed healing of the wound or even death of a patient because of distribution of integrity of the skin and immune system as well as poor self-care (32). Opportunistic fungi may invade deep into the wounds and contribute to delayed wound healing in some of the patients with diabetes who are otherwise immunocompromised when compared to the group without diabetes (11). If these infections are not noticed in time, these fungi colonize and damage the integrity of the skin, cause secondary infection through fissures, and increase the risk of soft tissue infections on the affected extremities. Although there is no evidence of fungal infection in the skin and nails of the patients, the infection is thought to develop after colonization. 

Mold infections are less common but more progressive than *Candida* spp. However, factors such as uncontrolled diabetes, presence of other underlying diseases, trauma, poor foot care, and recurrent infections may predispose the development of mold infections. There are only a few case reports that present causative fungal pathogens: calcaneal osteomyelitis is caused by *Aspergillus ochraceus* and infections of flexor digitorum-peroneal tendon sheaths by *Blastomyces *(33,34). Osteomyelitis is caused by the *Fusarium* species, filamentous fungi present as saprophytes in soil and animals, was first reported in a patient with diabetes by Bader et al. (30). *Fusarium* spp. is also the cause of onychomycosis (30,35). It is stated that osteomyelitis caused by the progression of skin and soft tissue infections may be originated from patient’s onychomycosis. Skin and nails are the main portals of entry, and cutaneous infections often develop after skin breakdown at the infection site and are present as necrotic lesions, cellulitis, chronic ulcers, and abscesses (30). In this study, only three patients had mold infections and the *Fusarium* species were causative; although one of the patients was amputated, the other two recovered after the antifungal treatment. Since the cases in this study were evaluated retrospectively, the presence of any fungal skin lesion or onychomycosis was not known before the diagnosis of diabetic foot infection.

The proper management of DFI needs appropriate antibiotic selection (22). Despite a proper surgical and antibacterial therapy, the outcome of patients could be poor and wound healing may be delayed up to 18 weeks (24,36,37). Several pathogenic abnormalities from intrinsic flaws in blood supply, angiogenesis, and matrix turnover to extrinsic factors such as infections and continued failure of recovery lead to diabetic foot ulcers trauma (11). 

Chellan et al. (13) mentioned the importance of studying the pathogenicity of fungi in deep tissues of DFI and their possible contribution to delayed wound healing. They also revealed that the use of antifungal therapy besides antimicrobial therapy for invasive fungal infections in diabetic foot not only reduces the wound size rapidly but also leads the way to complete healing. It was shown that fluconazole and standard treatments were more effective than the standard treatment alone in diabetic foot patients with invasive fungal infections in terms of wound size reduction and complete wound healing. Hence, it is important to investigate all specimens from DFI for the presence of fungi in addition to bacteria. These patients should be treated with culture-specific antifungal agents along with antibacterial agents (13,21). Heald et al. (14) mentioned that the *Candida* species is associated with long term nonhealing wounds in diabetic foot which heals after systemic antifungal therapy.

In conclusion, it is important to suspect invasive fungal infections in patients with uncontrolled glucose levels and in patients unresponsive to antibacterial treatment. Although rare, invasive fungal infections can cause diabetic foot infections long before the detection of neuropathy and vasculopathy, which are first tested in order to prevent foot infections. Early detection of fungal infections in high‑risk individuals is critical for the prevention of severe consequences such as foot amputation. Diabetic foot infections require the utmost attention and a multidisciplinary foot care team including an infectious disease specialist and a medical microbiologist. 

## Acknowledgments

We all thank Hilmi Güngör, Halit Özyalçın, and İdil Ünal for their kind interest.
